# The Beat

**DOI:** 10.1289/ehp.120-a192b

**Published:** 2012-05-01

**Authors:** Erin E. Dooley

## Banned Antibiotic in Poultry Feed

The U.S. FDA banned the use of fluoroquinolone antibiotics in poultry production in 2005 in part due to increased resistance to the drugs among *Campylobacter* bacteria. But a new study finds evidence that these antibiotics are still being used or are inadvertently contaminating poultry feed.^^1^^ The authors analyzed samples of U.S. and Chinese feather meal, a by-product of poultry production used in animal feed and as a fertilizer, and found the banned drugs in 60% of the U.S. samples. All the samples contained between 2 and 10 antibiotic residues, some at high enough levels to select for resistant bacteria during *in vitro* experiments.

## Gene–Environmental Interaction for Congenital Scoliosis?

In humans the *HES7* gene has been linked with congenital scoliosis, a spinal defect that occurs in about 1 in 1,000 live births. Now researchers using a mouse model have linked maternal hypoxia during pregnancy plus the presence of only one functioning copy of the *Hes7* gene with up to a 10-times-greater risk of congenital scoliosis in offspring compared with the genetic risk factor alone.^^2^^ Hypoxia, or inadequate oxygenation of tissues or the whole body, can be caused by active or passive smoking, high altitude, and use of certain prescription or recreational drugs.

**Figure:**
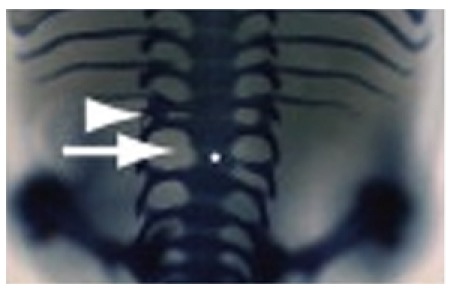
Moderate spinal abnormalities associated with hypoxia and haploinsufficiency of Hes7. © American Chemical Society/Cell

## Canadian Government Issues Draft Triclosan Assessment

On 31 March 2012 the Canadian government released its preliminary assessment of triclosan, a preservative and antimicrobial used in a wide array of cosmetics and personal care products.^^3^^ The assessment concludes that although triclosan is not harmful to human health, it is entering the environment in high enough quantities to potentially “have an immediate or long-term harmful effect on the environment or its biological diversity.” Comments on the draft will be accepted through May 2012.

## “Phantoms” Help Obese Patients Receive Safer Medical Imaging

Most medical imaging equipment is designed for normal-weight patients. Obese patients may be exposed to more radiation during computed tomography scans because their thicker layers of fat cause images to blur at normal radiation settings. In fact, obese men can receive 62% more radiation than their normal-weight counterparts, and obese women can receive 59% more, according to a new study.^^4^^

The study authors have developed realistic 3-D “phantoms,” or computer models of obese men and women that can help technicians calculate how to achieve the clearest images of such patients at the lowest radiation doses. The models will be part of a new software package that creates phantoms based on patients’ physical characteristics. These individualized phantoms could also help physicians track patients’ doses of radiation over their lifetimes, which is now required under California law.^^5^^

## Gasoline Engines May Outrun Diesel on SOA Production

Contrary to expectations, emissions from gasoline engines—not diesel engines—may contribute the most to the formation of secondary organic aerosols (SOAs), accounting for up to 80% of these airborne particles, according to a new estimate.^^6^^ In a study of Los Angeles air, researchers found that, although diesel emissions dipped by just over half on the weekends, SOA concentrations remained largely unchanged throughout the week. SOAs are linked to adverse respiratory and cardiovascular health effects. They also reduce visibility and play a poorly understood role in climate processes.
